# Protection of Mcc950 against high-glucose-induced human retinal endothelial cell dysfunction

**DOI:** 10.1038/cddis.2017.308

**Published:** 2017-07-20

**Authors:** Yi Zhang, Xuehua Lv, Zizhong Hu, Xiaojian Ye, Xinhua Zheng, Yuzhi Ding, Ping Xie, Qinghuai Liu

**Affiliations:** 1 Department of Ophthalmology, The First Affiliated Hospital of Nanjing Medical University, Nanjing 210029, China; 2 Department of Ophthalmology, The First People’s Hospital of Changzhou and The Third Affiliated Hospital of Soochow University, Changzhou 213003, China

## Abstract

Diabetic retinopathy (DR) is a well-known microvascular complication related to inflammation. Mcc950 is a potent and specific inhibitor of the NLRP3 inflammasome but its influence on DR has not been studied. Thus, we evaluated the anti-inflammatory effects of Mcc950 on high-glucose-induced human retinal endothelial cells (HRECs) and the potential underlying mechanism. In surgical excised proliferative membranes from DR patients, high expression of NLRP3, caspase 1 and IL-1*β* was observed and co-localization of NLRP3 and IL-1*β* occurred in CD31+ labeled HRECs. Moreover, in high-glucose-stimulated HRECs, increased production of the NLRP3 inflammasome activation and severe apoptosis were rescued with Mcc950 treatment. Additionally, the inhibitory effect of Mcc950 was mimicked through downregulation of NEK7 by siRNA in high-glucose-induced HRECs and Mcc950 treatment remarkably inhibited Nek7 and NLRP3 interactions by co-immunoprecipitation, suggesting that Mcc950 may be a potentially protective agent against inflammation, likely via downregulation of the Nek7-NLRP3 pathway. In conclusion, Mcc950 inhibited HREC dysfunction under high-glucose conditions and this research may offer insight for future pharmaceutical approaches for treating DR.

Diabetic retinopathy (DR) is a major diabetic complication that can cause significant visual impairment and blindness.^1, 2^ During the development of DR, retinal endothelial cell (REC) dysfunction is an important initiator of a multifactorial pathology^3, 4, 5^ that relies on metabolic abnormalities and inflammation.^6, 7^ Emerging evidence indicates that high-glucose-induced sterile inflammation is involved in the onset and progression of REC damage^8,9, 10, 11^; however, no effective drugs are available to treat REC damage.

The NLRP3 inflammasome, which is made up of NLRP3, apoptosis-associated speck-like domain containing a caspase-recruitment domain (ASC) and pro-caspase 1, is a group of intracellular innate immune proteins thought to act as sensors of pathogen- and damage- associated molecular patterns.^12^ The activation of NLRP3 inflammasome can promote the cleavage of pro-caspase 1 and pro-IL-1*β* into their mature pro-inflammatory forms,^13^ which contribute to the development of type 2 diabetes,^14^ atherosclerosis,^15^ gout,^16^ Alzheimer disease^17^ and age-related macular degeneration.^18^ Glucose abnormalities have also been reported to be an important trigger of the sterile inflammatory response which is mediated by the NLRP3 inflammasome.^6, 19^ Therefore, the NLRP3 inflammasome may be an effective target of pharmacological therapy to treat REC damage induced by high glucose.

Recently, a diarylsulfonylurea-based compound Mcc950 (or CRID3) was reported to be a highly specific NLRP3 inhibitor^20^ and its molecular target is of interest. Coll’s group^21^ reported that Mcc950 suppressed the formation of ASC complexes instead of blocking K^+^ efflux, Ca^2+^ flux or NLRP3–ASC interactions in response to NLRP3 and AIM2 stimulation. Also, Mcc950 specifically inhibits NLRP3 activation most likely by direct action on the protein or by interaction with pathways closely linked to NLRP3 activation.^20^ NEK7,^22^ as a Ser/Thr kinase during mitotic cell division, has been consistently proved to be a factor required for NLRP3 inflammasome activation by three independent groups.^23, 24, 25^ As a switch between inflammasome activation and cell division, NEK7 was confirmed to bind directly to NLRP3. The interaction between NLRP3 and NEK7 depends on LRR (leucine-rich repeats) or perhaps the NOD domain of NLRP3 and the kinase domain of NEK7.^22, 25^ Thus, NEK7-NLRP3 association is necessary for assembly and activation of the NLRP3 inflammasome. Furthermore, Shi’s group^23, 26^ suggests that Mcc950 or other glyburide-derived sulfonylureas might act by disrupting NEK7–NLRP3 interactions. We studied the role of Mcc950 in mediating high-glucose-induced NLRP3 inflammasome activity in human retinal endothelial cells (HRECs) and sought to identify whether the NEK7-NLRP3 pathway is involved in anti-inflammation effects of Mcc950.

## Results

### NLRP3 inflammasome activation in proliferative membranes from DR patients

**Figure 1:**
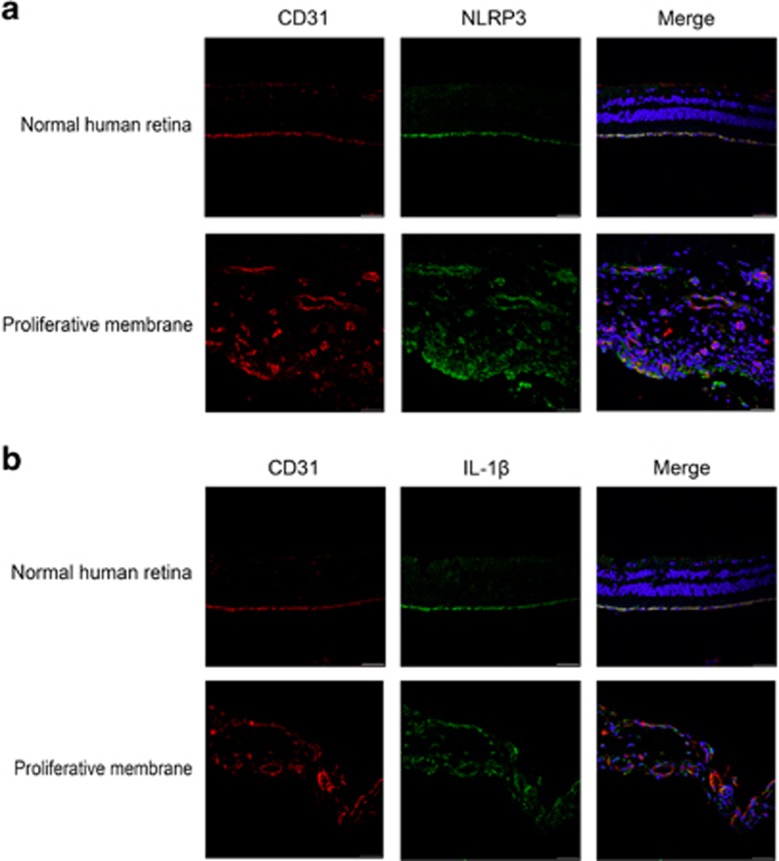
Immunofluorescence staining for NLRP3 inflammasome activation in normal human retinas and proliferative membranes from DR patients. (**a**) The co-localization of NLRP3 and CD31(endothelial cell adhesion molecule-1) in normal human retinas and proliferative membranes from DR patients by immunofluorescence (scale bar of 50 *μ*m). (**b**) The co-localization of IL-1*β* and CD31(endothelial cell adhesion molecule-1) in normal human retinas and proliferative membranes from DR patients by immunofluorescence

**Figure 2:**
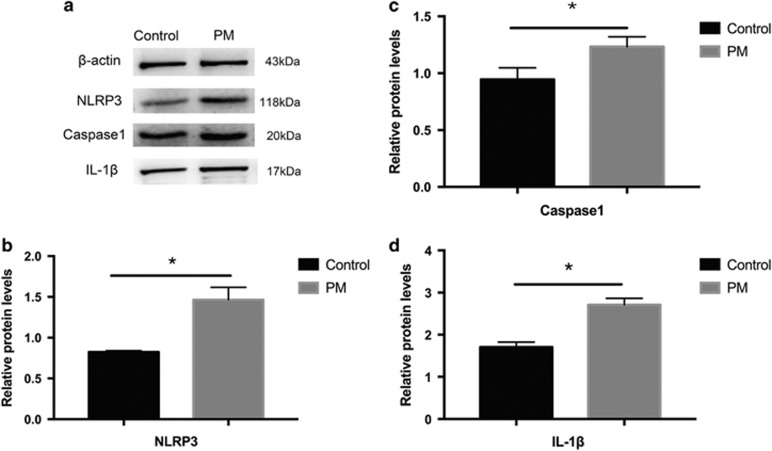
Western blot analysis of the expression of NLRP3, caspase1 and IL-1*β* in proliferative membranes. (**a**) The extracted protein was used to detect the expression levels of indicated proteins in proliferative membranes and normal human retinas. *β*-actin was used as protein loading control. (**b**–**d**): Relative expression level of NLRP3, caspase1 and IL-1*β* from three independent experiments was quantified. Expression is shown relatively to *β*-actin in each group, which was set to 1. Significant differences were calculated using Student’s *t*-test. *P<0.05 *versus* control. PM: proliferative membrane; Control: normal human retina

To learn whether the NLRP3 inflammasome is involved in the pathogenesis of membrane proliferation, we used immunohistochemistry with NLRP3, IL-1*β* and endothelial cell adhesion molecule 1 (CD31). Figure 1 shows more intense NLRP3 and IL-1*β* staining in CD31-labeled endothelial cells in proliferative membranes compared with controls. Additionally, western blot (Figure 2) confirmed elevated NLRP3, caspase 1 and IL-1*β* protein expression in proliferative membranes from DR and expression was modest in normal human retinas.

### Effect of high glucose or Mcc950 on HREC viability and proliferation

**Figure 3:**
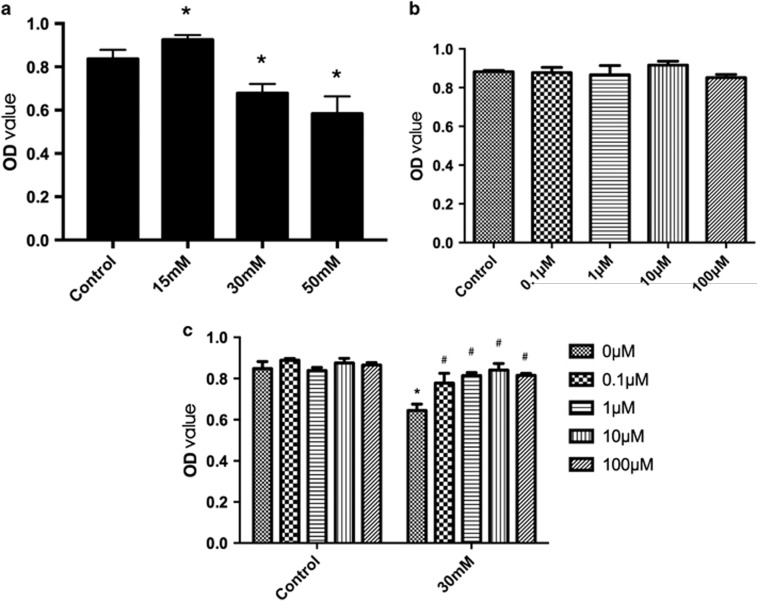
Effect of high glucose or Mcc950 on HREC viability and proliferation. (**a**) Dose–response effect of high glucose on HREC viability and proliferation. HRECs viability were tested in normal glucose condition (5.5 mM) or high glucose(15, 30, 50 mM) condition for 72 h. Data are shown as the mean±S.E.M., *n*=8 technical replicates. One-way ANOVA followed by Tukey’s multiple comparison test. *P<0.01 *versus* control. (**b**) Mcc950 (0.1,1,10,100 *μ*M) treatment for up to 48 h has no significant effect on cell viability and proliferation. Data are shown as the mean±S.E.M., *n*=3 technical replicates. One-way ANOVA followed by Tukey’s multiple comparison test. (**c**) Effect of Mcc950 on HRECs treated with 30 mM high glucose for 72 h. Cell survival shows significantly rescued by Mcc950 (0.1,1,10,100 *μ*M) in high-glucose condition. Data are shown as the mean±S.E.M., *n*=8 technical replicates. One-way ANOVA followed by Tukey’s multiple comparison test. *P<0.01 *versus* control, ^#^*P*<0.01 *versus* 30 mM high-glucose group

Figure 3a shows that compared with controls (5.5 mM normal glucose treatment), HRECs significantly increased after 15 mM high-glucose treatment for 72 h while approximately 18.1 and 28.7% reduction were induced after 30 mM and 50 mM high-glucose treatment, respectively. Because 50 mM glucose treatment may have produced unreliable data, 30 mM was used for subsequent experiments to study the protective effect of Mcc950 on high-glucose-induced cell death. Mcc950 (0.1, 1, 10, 100 *μ*M) had no significant effect on cell viability and proliferation (Figure 3b). However, pretreatment with Mcc950 rescued HREC viability in response to high-glucose stimulation (Figure 3c).

### Attenuation of high-glucose-mediated IL-1*β* secretion by Mcc950 pretreatment in HRECs

**Figure 4:**
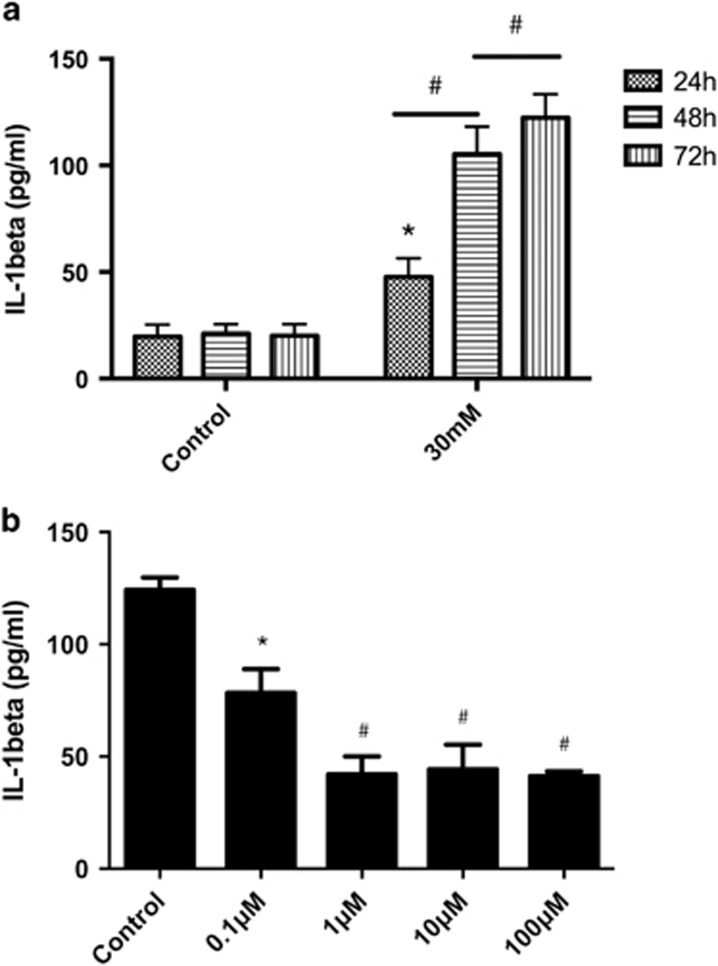
Mcc950 pretreatment attenuated high-glucose-mediated IL-1*β* secretion. (**a**) Measurement of IL-1*β* released from HRECs incubated in a media that contained 5.5, 30 mM glucose for 24,48 or 72 h, respectively. Data are shown as the mean±S.E.M., *n*=3 technical replicates. One-way ANOVA followed by Tukey’s multiple comparison test. *P<0.01 *versus* control, ^#^*P*<0.01 *versus* 24 h high-glucose treatment. (**b**) Concentration-dependent effect of Mcc950 on HRECs in 30 mM glucose condition for 72 h. Data are shown as the mean±S.E.M., *n*=3 technical replicates. One-way ANOVA followed by Tukey’s multiple comparison test. *P<0.05, ^#^*P*<0.01 *versus* control

A correlation between IL-1*β* released in cell supernatant with high-glucose exposure was studied and we found that IL-1*β* expression was increased after high-glucose exposure in a time- and concentration-dependent manner (Figure 4a and Supplementary Figure 4a). Figure 4b shows that more IL-1*β* was inhibited (but this was not significantly different among these three groups) in cells treated with 1, 10 and 100 *μ*M Mcc950 compared to 0.1 *μ*M Mcc950 or controls with 30 mM high glucose for 72 h. Therefore, 1 and 10 *μ*M Mcc950 were selected for subsequent experiments.

### Protection of Mcc950 against high-glucose-induced HREC apoptosis

**Figure 5:**
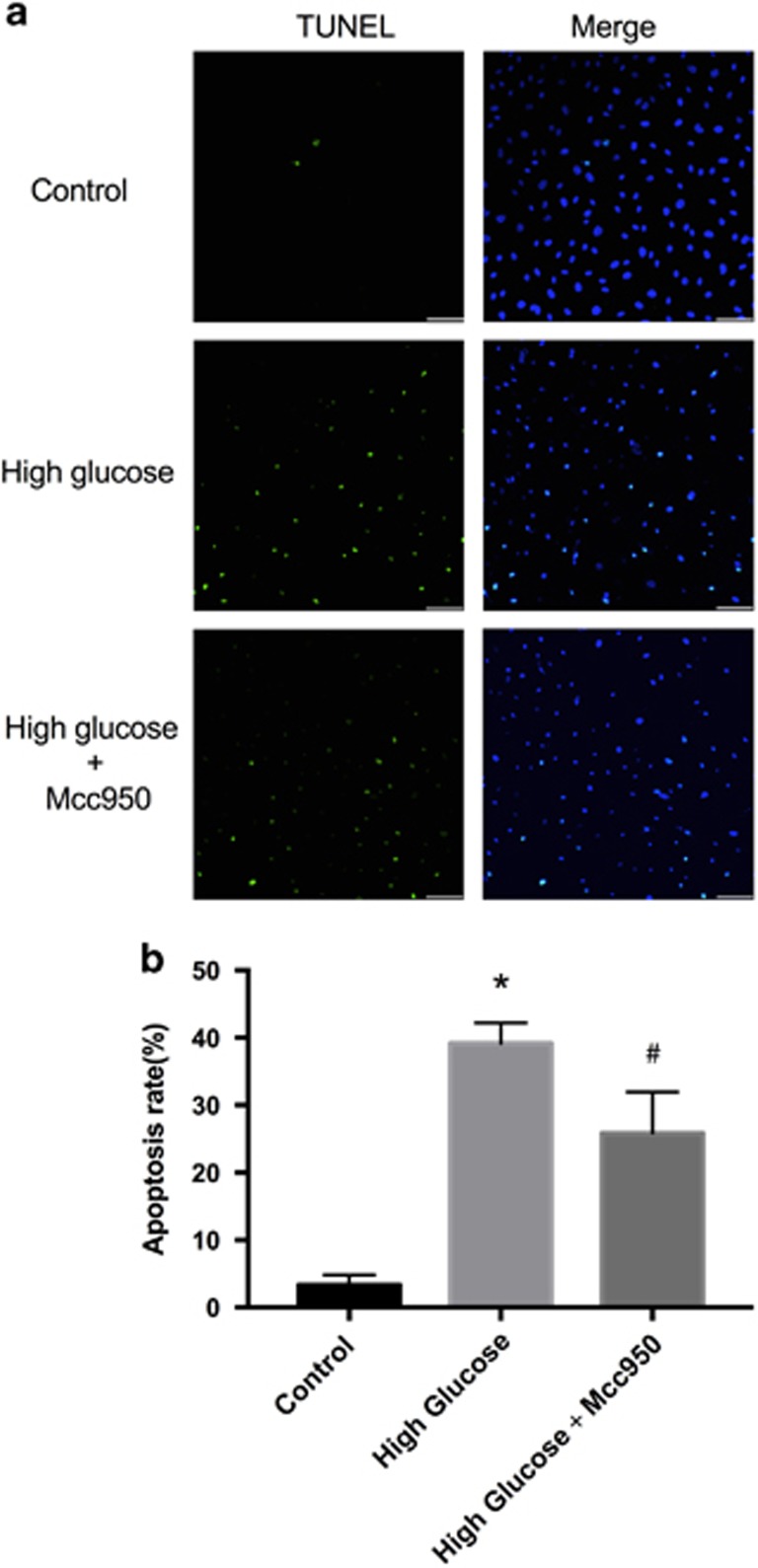
Protection effect of Mcc950 against high-glucose-induced HREC apoptosis. (**a**) Apoptosis cells in control culture medium, high-glucose culture medium or high glucose with Mcc950 culture medium were detected by TUNEL assay (scale bar of 50 *μ*m). (**b**) Quantitative analysis of TUNEL-positive cells after incubating in three different culture media. Data are shown as mean±S.E.M., *n*=4 technical replicates. Significant differences were calculated using one-way ANOVA followed by Tukey’s multiple comparison test. *P<0.01 *versus* control. ^#^*P*<0.01 *versus* high-glucose group

HREC apoptosis was measured and Figure 5a shows that few TUNEL-stained cells were apparent in controls (~3.2%) and more intense staining occurred with high glucose (~38.2%). Mcc950 significantly reduced the percentage of apoptotic cells to about 28.5% (Figure 5b) and data suggest the presence of an anti-apoptotic role of Mcc950 against NLRP3 inflammasome activation.

### Effect of Mcc950 on high-glucose-stimulated NLRP3 inflammasome mRNA expression in HRECs

**Figure 6:**
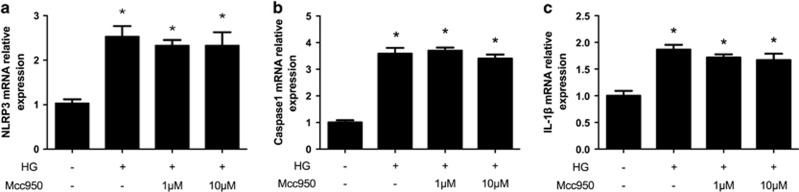
Effect of Mcc950 on high-glucose-stimulated NLRP3 inflammasome mRNA expression in HRECs. (**a**–**c**) Relative mRNA expression level of NLRP3, caspase1 and IL-1*β* was quantified. Expression is shown relatively to *β*-actin in each group, which was set to 1. Data are shown as mean±S.E.M., *n*=3 technical replicates. Significant differences were calculated using one-way ANOVA followed by Tukey’s multiple comparison test. *P<0.01 *versus* control

Transcript expressions (mRNA) of NLRP3, caspase 1 and downstream IL-1*β* in HRECs were obtained and NLRP3, caspase 1 and IL-1*β* mRNA were significantly increased compared to controls (Figures 6a–c). However, there were no significant differences between 1 or 10 *μ*M Mcc950 treatment groups and the high-glucose group.

### Mcc950 inhibited high-glucose-stimulated NLRP3 inflammasome activation in HRECs

**Figure 7:**
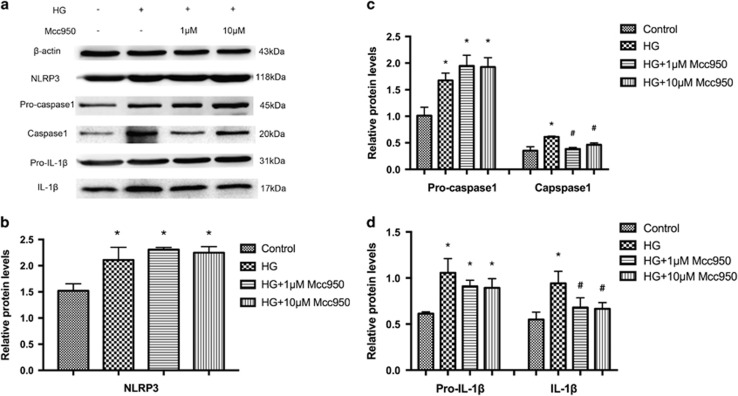
Mcc950 inhibited the activation of NLRP3 inflammasome by western blot analysis. (**a**) Expression level of indicated proteins was analyzed after incubating in normal culture medium, high-glucose culture medium, high-glucose culture medium with 1 *μ*M or 10 *μ*M Mcc950 supplementation. (**b**–**d**) Expression is shown relatively to *β*-actin in each group, which was set to 1. Data are shown as mean±S.E.M., *n*=3 technical replicates. Significant differences were calculated using one-way ANOVA followed by Tukey’s multiple comparison test. *P<0.05 *versus* control, ^#^*P*<0.05 *versus* high-glucose group. HG, high glucose

We measured post-transcriptional expression of multiple proteins in cell lysis buffer after high-glucose treatment with/without Mcc950 interference. Compared to controls, NLRP3, pro-caspase 1 and pro-IL-1*β* protein was increased after stimulation by high glucose and this high expression was sustained with 1 or 10 *μ*M Mcc950, which was consistent with mRNA expression data. However, mature caspase 1 and IL-1*β* were inhibited by the addition of 1 or 10 *μ*M Mcc950. Mcc950 suppressed activation of the NLRP3 inflammasome, sequentially disturbing the transformation of pro-caspase 1 and pro-IL-1*β* into mature caspase 1 and IL-1*β* (Figures 7a–d).

**Figure 8:**
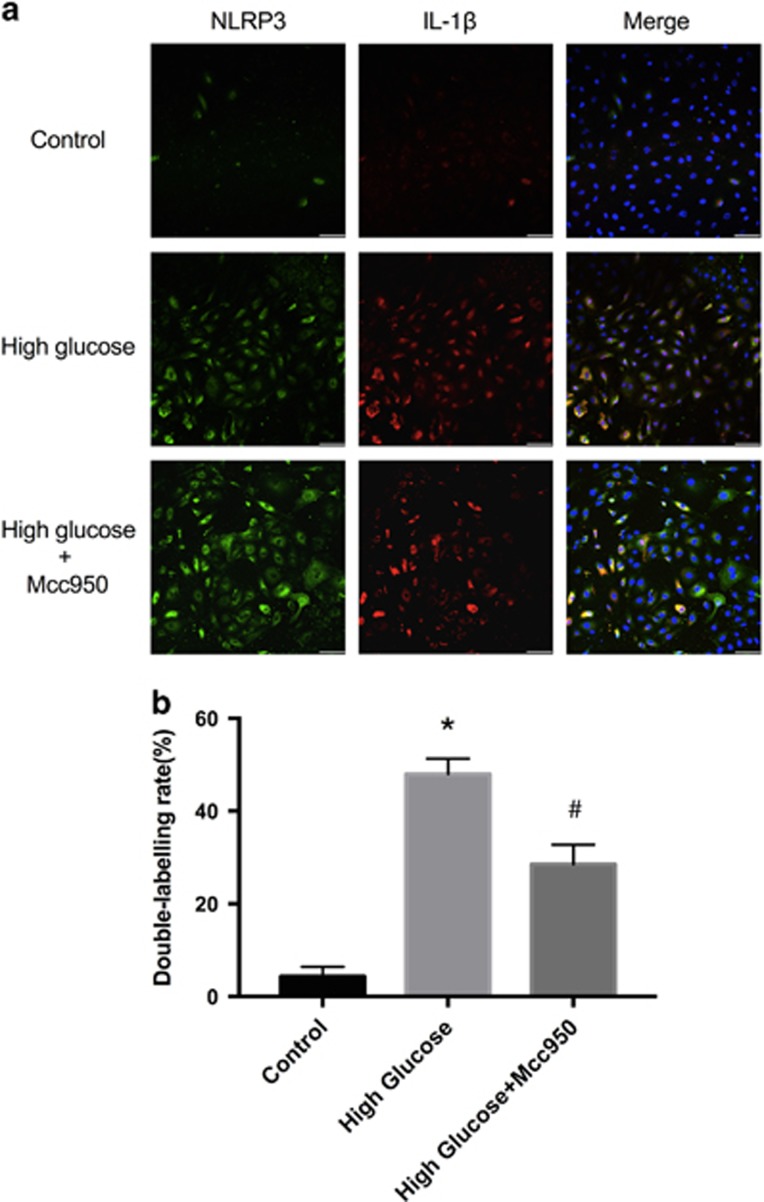
Immunofluorescence analysis of the expression of NLRP3 and IL-1*β* in HRECs. (**a**) Representative immunostaining images showed the NLRP3/ IL-1*β* double labeling HRECs induced by three different culture media (scale bar of 50 *μ*m). (**b**) Quantitative analysis of the ratio of NLRP3/IL-1*β* co-labeling cell to total cell after incubating in three different culture media. Data are shown as mean±S.E.M., *n*=3 technical replicates. Significant differences were calculated using one-way ANOVA followed by Tukey’s multiple comparison test. *P<0.01 *versus* control, ^#^*P*<0.05 *versus* high-glucose group

A subsequent analysis of the effect of Mcc950 on HRECs after stimulation with high glucose was made using immunofluorescence. Representative immunofluorescent images (Figure 8 and Supplementary Figure 8) indicate strong double-labeling of NLRP3 and IL-1*β* in the high-glucose group and that Mcc950 reduced the expression of IL-1*β*.

### NEK7 by siRNA inhibited high-glucose-stimulated NLRP3 inflammasome activation in HRECs

**Figure 9:**
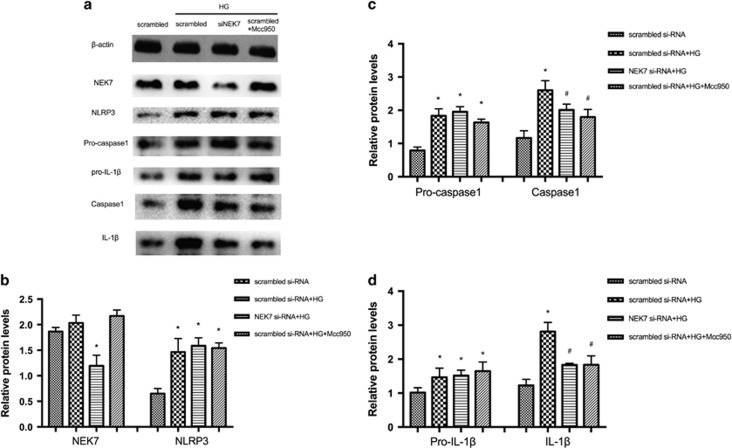
NEK7 by siRNA inhibited high-glucose-stimulated NLRP3 inflammasome activation in HRECs. (**a**) Expression level of indicated proteins in scrambled siRNA group, scrambled siRNA+ HG group, NEK7 siRNA+ HG group, scrambled siRNA+HG+Mcc950 group was detected by western blot, respectively. (**b**–**d**): Expression is shown relatively to *β*-actin in each group, which was set to 1. Data are shown as mean±S.E.M., *n*=3 technical replicates. Significant differences were calculated using one-way ANOVA followed by Tukey’s multiple comparison test. *P<0.05 *versus* scrambled siRNA group, ^#^*P*<0.05 *versus* scrambled siRNA+ HG group

To explore whether NLRP3–NEK7 interactions contribute to HRECs induced by high glucose, we measured changes in expression of the NLRP3 inflammasome in HRECs transfected with si-NEK7. Figure 9 shows that NEK7 protein expression was reduced by si-NEK7 but there was no significant change with high glucose or Mcc950 treatment. Compared with scrambled siRNA group with no treatment, NLRP3, pro-caspase 1, pro-IL-1*β*, caspase 1 and IL-1*β* protein increased after stimulation with high glucose in the scrambled siRNA group, the si-NEK7 group and in the 10 *μ*M Mcc950-treated group. However, caspase 1 and IL-1*β* protein were suppressed in the si-NEK7 group and Mcc950-treated group. Thus, depletion of NEK7 and addition of Mcc950 inhibited NLRP3 inflammasome activation induced by high glucose in HRECs.

### Anti-inflammatory effect of Mcc950 via NLRP3–NEK7 pathway in high-glucose-stimulated HRECs

**Figure 10:**
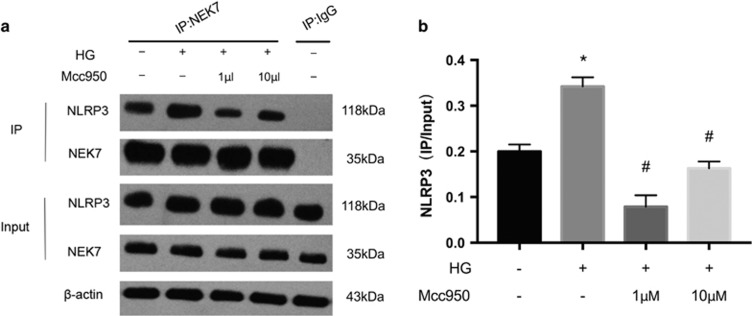
Mcc950 suppressed high-glucose-induced HREC dysfunction via NLRP3–NEK7 pathway. (**a**) Effect of Mcc950 on the endogenous NEK7–NLRP3 interaction in high-glucose condition was examined by immunoprecipitation and immunoblot. The lysate of control group without HG or Mcc950 treatment was applied for IgG control, using the same amount of protein. (**b**) The density value of NLRP3 interacted with NEK7 was normalized for its input protein level. Data are shown as mean±S.E.M., *n*=2–3 technical replicates. One-way ANOVA followed by Tukey’s multiple comparison test. *P<0.01 *versus* control, ^#^*P*<0.01 *versus* HG group

Co-immunoprecipitation was used to examine the interaction between NEK7 and NLRP3 in the presence/absence of Mcc950. The NLRP3–NEK7 interaction was significantly enhanced after high-glucose stimulation, but was suppressed in both Mcc950 treatment groups (Figures 10a and b). Thus, there is an inhibitory effect of Mcc950 on NLRP3 inflammasome activation by the NEK7–NLRP3 signaling.

## Discussion

Activation of the NLRP3 inflammasome is thought to be key for the progression of pro-inflammatory effects of retinopathy including age-related macular degeneration^18^ and DR.^27^ Using immunofluorescence and western blot we confirmed NLRP3 inflammasome activation in human REC of proliferative membranes, a crucial characteristic of PDR.^28, 29, 30^ The anti-NLRP3 inflammasome effect of Mcc950 for macrophages, microglia, myoblasts and dendritic cells is well described,^31, 32, 33, 34^ but its effect on inflammation of HRECs is unexplored. Thus, we investigated the potency of Mcc950 for attenuating inflammation in HRECs induced by high glucose.

In HRECs we observed that high glucose upregulated inflammatory cytokine IL-1*β*, which induces production of oxygen radicals to damage DNA^35, 36^ and induce apoptosis.^37, 38^ Mcc950 not only inhibited NLRP3 inflammasome activation as evidenced by suppressing cleavage of caspase 1 and downstream interleukin IL-1*β* production, but decreased apoptosis and mature IL-1*β* released by HRECs. Thus, Mcc950 has an anti-inflammatory role in the pathogenesis of impaired glucose-mediated retinopathy, and these data agree with previous reports that Mcc950 can inhibit NLRP3 inflammasome activation in dermal and pulmonary inflammation,^31^ crystal-induced kidney fibrosis,^33^ valosin-containing protein-associated diseases,^32^ cryopyrin-associated periodic syndromes,^20, 39, 40^ hypertension,^41^ and myocardial infarction.^42^

We measured NLRP3, pro-caspase 1 and pro-IL-1*β* mRNA before and after Mcc950 treatment in high-glucose-stimulated HRECs, and found no significant difference in either setting, consistent with data from Perregaux’s group.^20, 43^ Interestingly, mature caspase 1 and IL-1*β* protein expression significantly reduced while NLRP3, and pro-caspase-1 and pro-IL-1*β* were still highly expressed after Mcc950 treatment with high glucose. Thus, according to Coll and Petra Sušjan’s theory,^20, 44^ Mcc950 does not directly target NLRP3 inflammasome activation at the genetic level but rather influences protein interaction or pathways closely linked to NLRP3 activation. Of note, two proteins, GSTO1^45^ and Txnip,^46^ have initially been considered to be possible targets of Mcc950. However, co-immunoprecipitation experiments indicated that GSTO1 interacted with ASC rather than NLRP3 directly.^21^ Although the thioredoxin interacting protein (Txnip) was identified as a redox sensitive ligand of NLRP3,^46^ not enough evidence suggested that the signaling pathway leading to NLRP3 activation requires Txnip.^47^

NEK7 has been confirmed to bind directly to NLRP3 and is a factor required for NLRP3 inflammasome activation in macrophages induced by nigericin.^23^ In the present study, we observed that downregulation of NEK7 by siRNA inhibited NLRP3 inflammasome activation, which partly mimicked the effect of Mcc950 treatment in high-glucose-induced HRECs. However, there was no significant change in expression of NEK7 with the addition of Mcc950. We next verified whether Mcc950 can target the interaction between NEK7 and NLRP3 and co-immunoprecipitation data showed that the NEK7–NLRP3 interaction responded to high glucose stress as well as LPS priming after ATP stimulation,^23^ but this was suppressed by Mcc950. This is the first confirmation of the hypothesis of Shi’s group^23, 26^ that the inhibitory effect of Mcc950 on NLRP3 inflammasome activation is partly mediated by downregulating NEK7–NLRP3 activity. Thus, Mcc950 can protect HRECs from high-glucose-induced dysfunction via disrupting the binding of NEK7 with NLRP3, and therefore, Mcc950 may be a promising pharmaceutical approach for the future treatment of DR.

## Materials and methods

### Human surgical samples

All procedures abided by the tenets of the Declaration of Helsinki (for human subjects) and to the ARVO Statement on human subject research. We received institutional approval from the review committee of The First Affiliated Hospital with Nanjing Medical University. Proliferative membranes were surgically removed from six patients with PDR (ages 60±6 years; duration of diabetes 16±7 years), who underwent *pars plana* vitrectomy and membrane peeling by the same surgeon, and retinal sections from normal donor eyes (*n*=4) were used as controls (aged 40±5 years).

### Cell culture and siRNA-mediated interference

HRECs were obtained from Angioproteomie company (Boston, MA, USA). HRECs were cultured in endothelial cell medium (Sciencell, Carlsbad, CA, USA) supplemented with 5% fetal bovine serum (FBS), 100 *μ*g/ml penicillin, and 100 *μ*g/ml streptomycin (Gibco Laboratories, Grand Island, NY, USA). HRECs were incubated at 37 °C in a humidified atmosphere containing 5% CO_2_ and air. A separate cohort of HRECs was exposed to normal glucose (NG, 5.5 mM D-glucose (Sigma, St. Louis, MO, USA)), 30 mM high glucose and 50 mM high glucose in the presence or absence of Mcc950 (Tocris Bioscience, Bristol, UK). Experiments were performed between cell passages 3 and 8.

Nek7-specific siRNA (siNek7) and scrambled siRNA (siScrambled) were purchased from (GenePharma, Shanghai, China). HRECs (60–70% confluent) were transfected with Lipofectamine 2000 Reagent (Invitrogen, Carlsbad, CA, USA) at a final siRNA concentration of 100 nmol/l. Six hours after transfection with the indicated concentrations, media was replaced with fresh culture medium and the transfected cells were incubated for 48 h. Oligonucleotides used for Nek7 were: siNek7: 5′-AUAUUAACUAACUGUCGGAGdTdT-3′ and control scrambled-siRNA: 5′-GCACUAACCUACCAACAAUdTdT-3′.

### Cell viability assay

Viability and proliferation of HRECs was measured using a cell counting kit-8 (CCK-8, Biosharp, Hefei, China). Briefly, 2 × 10^3^ of HRECs were seeded into each well of a 96-well plate and allowed to attach for 24 h. Cells were cultured in serum-free media for starvation for 12 h. Then, cells were stimulated with different concentrations of glucose with or without Mcc950 (0.1, 1, 10, 100 *μ*M) for 24 h, and 10 *μ*l CCK-8 was added to each well followed by incubation for an additional 2–4 h at 37 °C. Then, absorbance (450 nm) was measured. Experiments were repeated at least three times.

### Western blot

HRECs obtained from passage 6 were grown to 70–80% confluence and then starved for 12 h in 0.5% FBS/ECM. HRECs were pretreated with Mcc950 for 2 h before stimulation with high glucose. Then, HRECs and proliferative membrane samples from DR patients or donor eyes were lysed using a nuclear and cytoplasmic protein extraction kit (Beyotime, Haimen, China). Lysates were centrifuged at 15 000 × *g* for 10 min at 4 °C. Protein was quantified using Bradford’s reagent with bovine serum albumin as a standard. Proteins were separated using 10% SDS-PAGE and transferred to a polyvinylidene difluoride membrane (Millipore, IPVH00010, Bedford, MA, USA). Then, the membrane was blocked for 1 h at room temperature with 5% (v/v) nonfat dry milk. After three washes with PBST, the membrane was incubated in PBS at 4 °C (overnight) with anti-caspase 1 (1:1000, Cell Signaling Technology, Boston, MA, USA, No.2225), anti-NLRP3 (1:1000, Proteintech, Chicago, USA, No. 19771-1-AP), anti-IL-1*β* (1:1,000, Cell Signaling Technology, No. 12242). The membrane was again washed with PBST and incubated for 1 h at room temperature with a horseradish peroxidase-conjugated secondary antibodies. Signals were developed using a standard ECL western blot detection reagent (Amersham Biosciences, Arlington Heights, IL, USA). Densitometric analysis was performed with ImageJ software.

### Co-immunoprecipitation assay

Cells were extracted with lysis buffer (10 mM KCl, 1.5 mM MgCl_2_, 10 mM HEPES, 1 mM PMSF, 1 mM DTT) and homogenized for 30 min at 4 °C. Protein extracts were centrifuged at 12 000 × *g* for 15 min at 4 °C, and then the supernatants containing total protein were collected. Equal amounts of protein were exposed to antibodies against control immunoglobulin G (1:100, Abcam, Cambridge, MA, USA, ab200699) or Nek7 (1:10, Abcam, ab133514), which was immobilized on protein A/G beads (Beyotime). After a 3 h incubation at 4 °C with gentle rotation, beads were washed extensively five times with lysis buffer, boiled and microcentrifuged. Proteins were detected with monoclonal antibodies against Nek7 (1:10 000, Abcam, ab133514), NLRP3 (1:1000, Proteintech, No. 19771-1-AP) by western blot.

### RNA isolation and cDNA synthesis

At the end of the time point, cells were trypsinized, washed with ice-cold PBS twice and total RNA was isolated using Trizol reagent (Life Technologies, Grand Island, NY, USA). RNA was measured using 260/280 UV spectrophotometry. Total RNA pellets were resuspended in RNase-free water, followed by removal of potentially contaminated DNA by treatment with DNase I (Life Technologies). Next, 1 *μ*g of total RNA from each sample was used for reverse transcription with an oligo-dT and a Superscript II (Life Technologies) to generate first-strand cDNA in a 20 *μ*l reaction mixture. Finally, the cDNA was stored at −20 °C before use.

### Real-time PCR

RT-PCR was performed to measure expression of mRNA with the following primers: human NLRP3 (forward, 5′-GCACCTGTTGTGCAATCTGAA-3′ reverse, 5′-TCCTGACAACATGCTGATGTGA)-3′, human capsase1 (forward, 5′-TTTCCGCAAGGTTCGATTTTCA-3′ reverse, 5′-TGGGCATCTGCGCTCTACCATC-3′), human IL-1*β* (forward, 5′-TCCAGGGACAGGATATGGAG-3′ reverse, 5′-TCTTTCAACACGCAGGACAG-3′). A 4.4 *μ*l aliquot of cDNA was amplified using RT-PCR in a total volume of 10 *μ*l. RT-PCR was performed with an initial denaturation step at 95 °C for 30 min followed by 40 cycles of standard PCR. NLRP3, caspase 1, IL-1*β* mRNA expression were normalized to *β*-actin mRNA.

### ELISA

ELISA was conducted on culture media collected after treatment. Media samples were immediately centrifuged for 5 min at 4000 × *g* to collect conditioned culture supernatant, which was stored at −80 °C until use. IL-1*β* released by HRECs was measured using a commercially available ELISA kit (R&D System, Minneapolis, MN, USA) according to the protocol described by the manufacturer.

### Immunofluorescence

Samples from patients with PDR or donor eyecups were fixed with freshly prepared paraformaldehyde (4%) for 2 h at 4 °C, dehydrated using 30% sucrose solutions for 30 min and sent immediately to OCT compound (Sakura, PA, USA) for frozen sections. Frozen sections were cut at 10 *μ*m thickness at −20 °C and stored at −80 °C until staining. Sections were stained by NLRP3 (1:500, Abcam, ab4207) or IL-1*β* (1:500, Abcam, ab2105) with CD31 (1:500, Abcam, ab24590). HRECs were fixed with freshly prepared paraformaldehyde (4%) for 30 min at 4^ ^°C. Then, cells and frozen tissue sections were washed 10 min with PBS (three times), and blocked with 1% FBS in PBS for 1 h at room temperature. After a 10-min washing with PBS three times, samples were incubated with NLRP3 or IL-1*β* overnight at 4 °C in a humidified chamber. After a 10-min washing with PBS three times, cells were incubated with corresponding secondary antibodies conjugated with Alexa Fluor 488 (1:500, Abcam) or Alexa 594 (1:500, Abcam) for 1 h at 37 °C in a darkened humidified chamber. Cell nuclei were stained with 4′,6-diamidino-2-phenylindole (DAPI, Sigma, St. Louis, MO, USA). Fluorescence was observed using a confocal microscope (Olympus 1X81, Olympus, Tokyo, Japan).

### TUNEL staining

Cell death was measured using TUNEL (Roche Biochemicals, Mannheim, Germany), according to the manufacturer’s instructions. After treatment with 4% freshly prepared paraformaldehyde for 30 min, samples were washed in PBS 10 min. Then, samples were treated with permeabilization solution (0.1% Triton X-100 in 0.1% sodium citrate) for 15 min. After washing, the labeling reaction was performed using a solution containing terminal deoxynucleotidyl transferase, its buffer and fluorescein-dUTP. During this step, slides were incubated at 37 °C for 60 min in a humidified chamber. After washing, cell nuclei were stained with DAPI for 15 min. Fluorescent images were photographed using a confocal microscope (Olympus 1X81, Olympus, Tokyo, Japan).

### Statistical analysis

All data are expressed as means±S.E.M. One-way ANOVA or a Student’s *t*-test was performed to assess statistical differences between the groups using SPSS19.0 software and Graphpad Prism 5.0 (*P*-value<0.05 was accepted as statistically significant).
