# Supplementary figures and images for: Protection of Mcc950 against high-glucose-induced human retinal endothelial cell dysfunction
Source: Cell Death Dis. 2017 Jul 20;8(7):e2941–. doi: 10.1038/cddis.2017.308 (PMC5550855; doi:10.1038/cddis.2017.308)

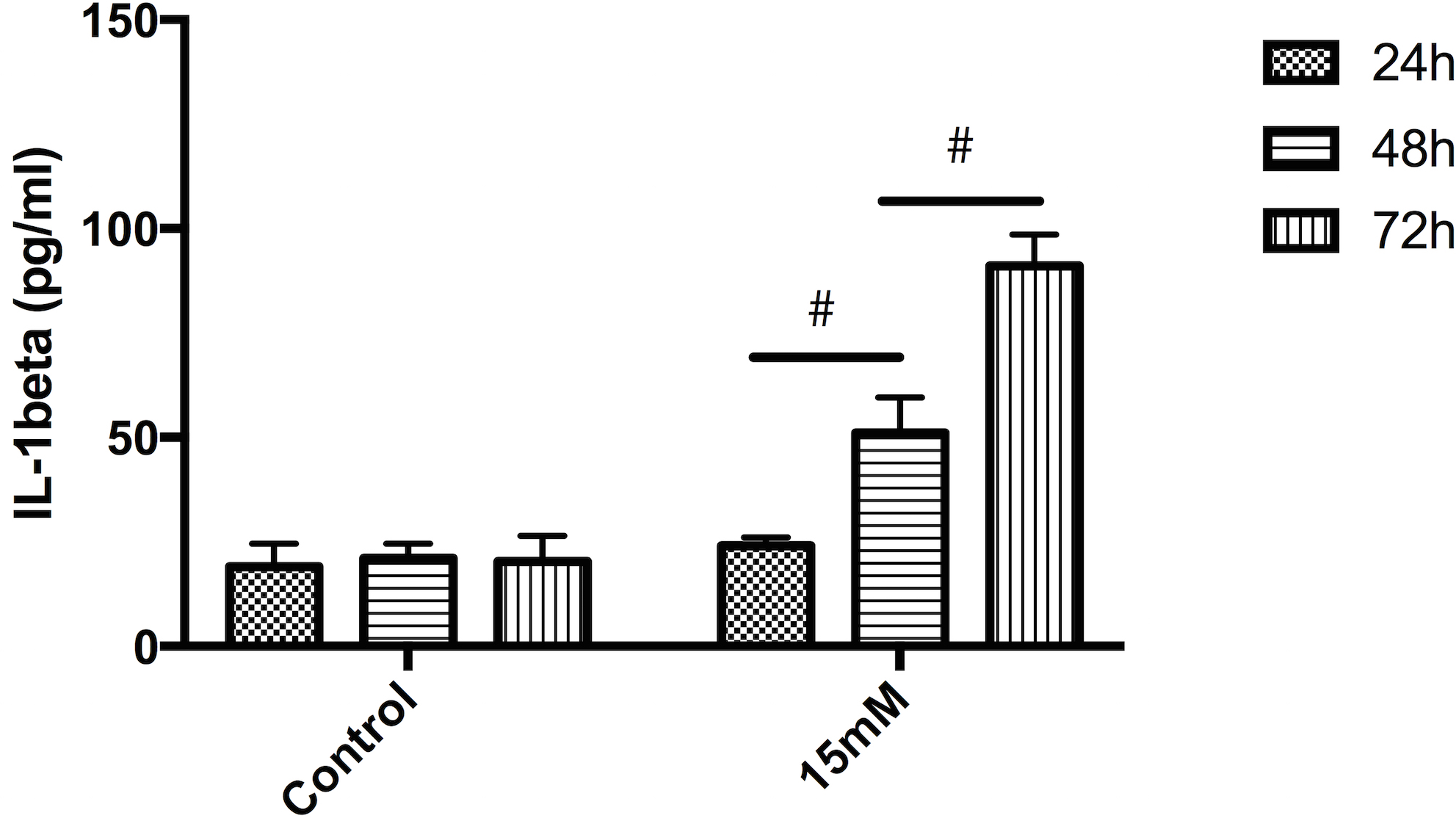

Supplement: Supplementary Figure 1 [file cddis2017308x1.tif]

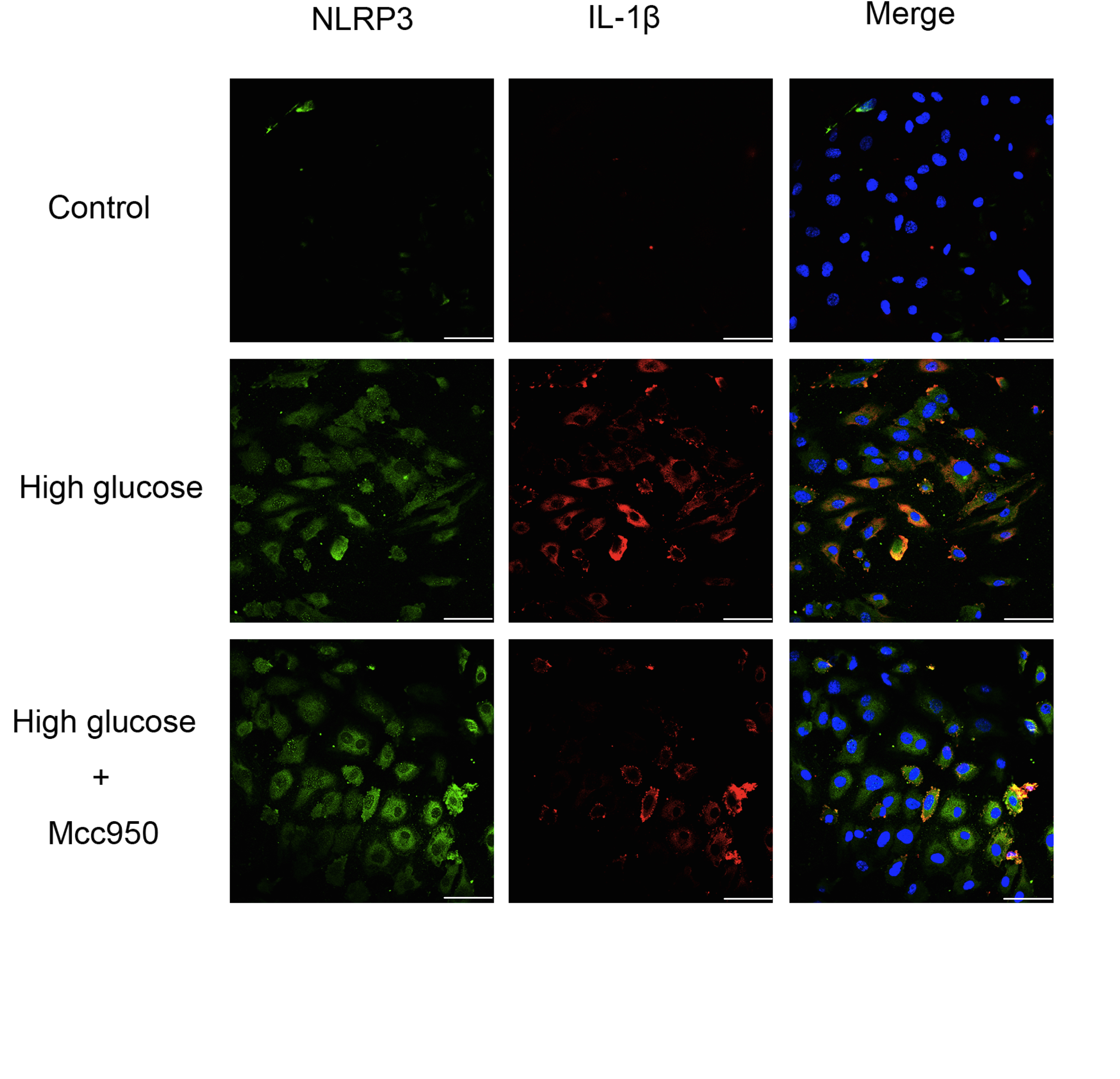

Supplement: Supplementary Figure 2 [file cddis2017308x2.tif]
